# Supplementary material for: Shape-directed rotation of homogeneous micromotors via catalytic self-electrophoresis
Source: Nat Commun. 2019 Jan 30;10:495. doi: 10.1038/s41467-019-08423-7 (PMC6353883; doi:10.1038/s41467-019-08423-7)
Supplement: Supplementary file 1 — Supplementary Information [file 41467_2019_8423_MOESM1_ESM.pdf]

Shape-directed rotation of homogeneous micromotors via catalytic self-electrophoresis.
Brooks *et al.*

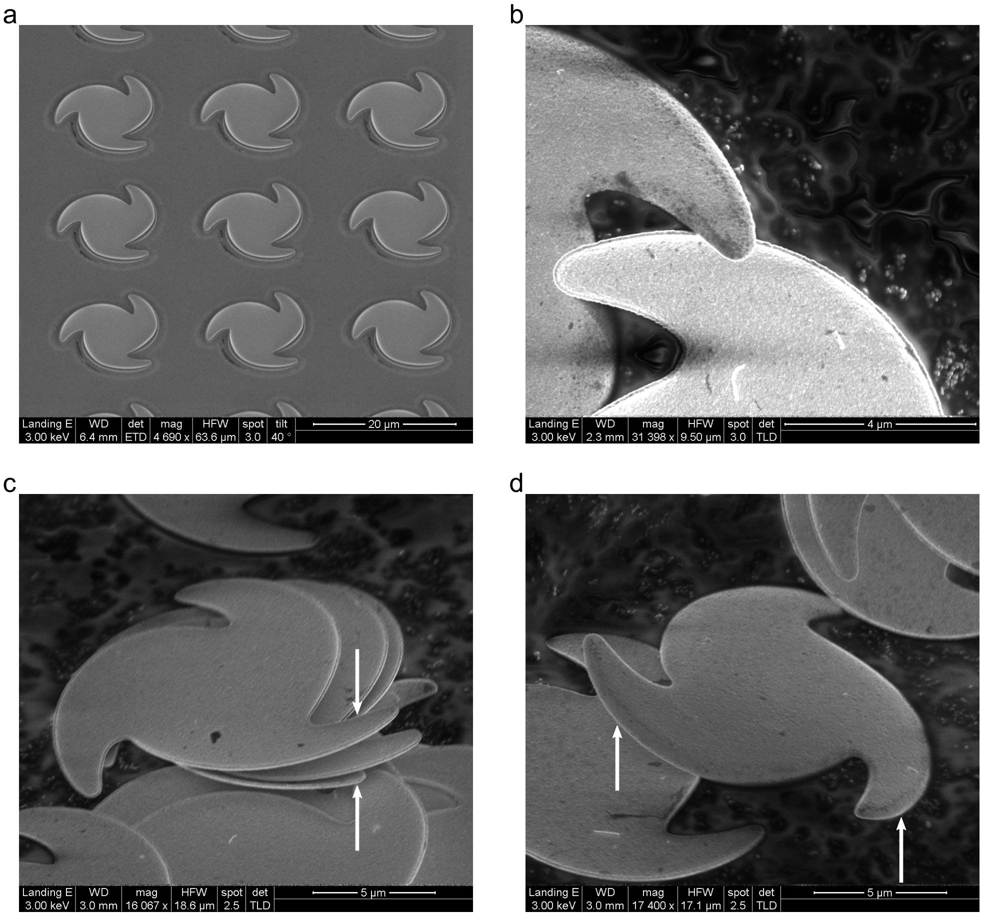

Supplementary Figure 1: Characterization of particle shape. Scanning electron microscopy images of Pt motors with number of fins $n = 3$, fin asymmetry $c = 2$, size $a = 5.77$ μm, fin length $b = 0.3$, and thickness $\delta = 100$ nm. Images were taken with a FEI Nova NanoSEM 630 FESEM. (a) Motors on silver-coated silicon wafer substrate. Motors were imaged after photolithography but prior to their removal from the substrate (see the "Fabrication of Platinum Motors" heading in the Methods section of the main text). (b) Texture of bottom surface (top left) and top surface (bottom right) of motors after removal from the silicon substrate. (c) and (d) Particles show slight bending, which leads to favorable settling in experiments.

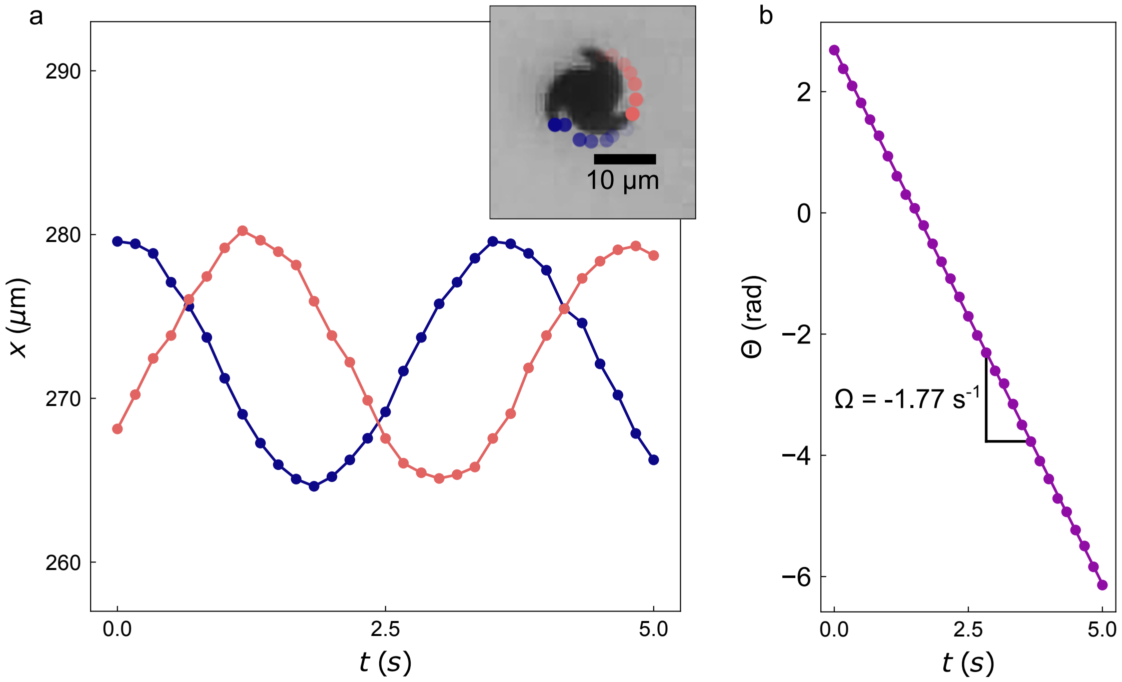

Supplementary Figure 2: Particle tracking and data analysis. Example of particle tracking methodology for a single particle with number of fins $n = 3$, fin asymmetry $c = 2$, size $a = 5.77$ μm, and fin length $b = 0.3$ in 10 wt % H_2O_2. (a) Measured position of a single particle over time. The coordinates of two points on the particle were tracked for 5 seconds at 6 frames per second. The inset shows an image of the particle taken by optical microscopy with the position of each tracked area highlighted. The plot shows the x-coordinate of both tracked areas. (b) Angular orientation vs. time for the same particle. Orientation was determined from the x- and y-coordinates of the two tracked areas on the particle. Data was fit to a line by the least squares method in order to determine the angular velocity of the particle.

Supplementary Note 1: Non-dimensionalization of the Model

The governing equations and boundary conditions outlined in the Methods can be non-dimensionalized using the following characteristic scales:

$$[C_i] = C_{\pm}^{\infty} \qquad \text{bulk ion concentration} \tag{1}$$
$$[\boldsymbol{x}] = \lambda \qquad \text{reaction-diffusion length} \tag{2}$$
$$[\phi] = k_B T/e \qquad \text{thermal potential} \tag{3}$$
$$[\boldsymbol{u}] = U_0 \qquad \text{characteristic velocity} \tag{4}$$

Here, the bulk ion concentration is related to the peroxide concentration as

$$C_{\pm}^{\infty} \equiv \sqrt{K_p C_{\mathrm{H_2O_2}}^0}. \tag{5}$$

The reaction-diffusion length λ is defined as

$$\lambda^2 \equiv \frac{D_{\pm}}{k_{-p} C_{\pm}^{\infty}}. \tag{6}$$

The characteristic diffusivity $D_{\pm}$ is defined as the average diffusivity of the dominant ions H^+ and HO_2^-

$$D_{\pm} \equiv \frac{1}{2}(D_{\mathrm{H^+}} + D_{\mathrm{HO_2^-}}). \tag{7}$$

Finally, the characteristic velocity U_0 is defined as

$$U_0 \equiv \frac{C_{\pm}^{\infty} k_B T \lambda}{\eta}. \tag{8}$$

In the subsequent sections, we reformulate the governing equations and boundary conditions using these scales and discuss the various dimensionless parameters that emerge. For simplicity of notation, we use the same symbols for dimensional and dimensionless variables alike; the distinction should be clear from the context.

Species Transport. The dimensionless concentration of species i is governed by the conservation equation

$$\nabla \cdot \boldsymbol{j}_i = \sum_j \nu_{ij} R_j \quad \text{with} \quad \boldsymbol{j}_i = \mathrm{Pe}\, \boldsymbol{u} C_i - D_i \nabla C_i + z_i D_i C_i \nabla \phi, \tag{9}$$

where $\mathrm{Pe} = U_0 \lambda / D_{\pm}$ is the Peclet number. We anticipate that the Péclet number will be very small such that convective species transport can be neglected. The boundary conditions far from the particle become

$$C_{\mathrm{H_2O_2}}(\boldsymbol{x}) = \gamma, \quad C_{\mathrm{H^+}}(\boldsymbol{x}) = C_{\mathrm{HO_2^-}}(\boldsymbol{x}) = 1, \quad C_{\mathrm{OH^-}}(\boldsymbol{x}) = 0, \quad \text{for } |\boldsymbol{x}| \to \infty, \tag{10}$$

where the dimensionless peroxide concentration is $\gamma = (C_{\mathrm{H_2O_2}}/K_p)^{1/2} \gg 1$. The dimensionless reaction rates are

$$R_w = 0 - C_{\mathrm{H^+}} C_{\mathrm{OH^-}}, \tag{11}$$
$$R_p = \frac{1}{\gamma} C_{\mathrm{H_2O_2}} - C_{\mathrm{H^+}} C_{\mathrm{HO_2^-}}. \tag{12}$$

The dimensionless current densities at the particle surface are given by

$$i_a = \mu C_{\mathrm{HO_2^-}} \exp\left(\alpha_a \Delta\Phi\right), \tag{13}$$

$$i_c = -\frac{\mu}{\gamma} C_{\mathrm{H_2O_2}} \exp\left(-(1-\alpha_c)\Delta\Phi\right), \tag{14}$$

where the parameter μ characterizes the relative rates of reaction and diffusion at the particle surface

$$\mu = \frac{k_a \lambda}{e D_\pm} \exp(\alpha_a \Phi_0) = \frac{k_c \lambda C^0_{\mathrm{H_2O_2}}}{e D_\pm C^\infty_\pm} \exp(-(1-\alpha_c)\Phi_0). \tag{15}$$

This equation defines the reference potential Φ_0 from which the Stern layer voltage is measured, $\Delta\Phi = \Phi_p - \Phi_s - \Phi_0$. This potential difference $\Delta\Phi$ must be determined self-consistently to ensure the condition of zero net current

$$\int_{\mathcal{S}} (i_a + i_c) \mathrm{d}\mathcal{S} = 0. \tag{16}$$

The boundary conditions at the particle surface become

$$i_a = -2(\boldsymbol{n} \cdot \boldsymbol{j}_{\mathrm{HO_2^-}}) = 2(\boldsymbol{n} \cdot \boldsymbol{j}_{\mathrm{H^+}}) \quad \text{for } \boldsymbol{x} \in \mathcal{S}. \tag{17}$$

$$i_c = 2(\boldsymbol{n} \cdot \boldsymbol{j}_{\mathrm{H_2O_2}}) = -(\boldsymbol{n} \cdot \boldsymbol{j}_{\mathrm{OH^-}}) \quad \text{for } \boldsymbol{x} \in \mathcal{S}, \tag{18}$$

Electrostatics. The dimensionless potential ϕ is governed by

$$-\nabla^2 \phi = \frac{1}{2\beta^2}(C_{\mathrm{H^+}} - C_{\mathrm{HO_2^-}} - C_{\mathrm{OH^-}}). \tag{19}$$

where $\beta = \lambda_D/\lambda$ is the ratio between the Debye screening length, $\lambda_D^2 = \varepsilon k_B T / 2e^2 C^\infty_\pm$, and the reaction-diffusion length λ. Far from the particle, the potential decays to zero; at the particle surface, we assume a constant surface potential Φ_s,

$$\phi(\boldsymbol{x}) = 0 \quad \text{for } |\boldsymbol{x}| \to \infty, \tag{20}$$

$$\phi(\boldsymbol{x}) = \Phi_s \quad \text{for } \boldsymbol{x} \in \mathcal{S}. \tag{21}$$

Hydrodynamics. The dimensionless velocity and pressure are governed by the Stokes equations including an electric body force $\boldsymbol{f}_e$

$$\nabla \cdot \boldsymbol{u} = 0 \tag{22}$$

$$0 = -\nabla p + \nabla^2 \boldsymbol{u} + \boldsymbol{f}_e \quad \text{with} \quad \boldsymbol{f}_e = -\nabla\phi \sum_i z_i C_i. \tag{23}$$

The boundary conditions at the particle surface $\mathcal{S}$ and far from the particle are

$$\boldsymbol{u}(\boldsymbol{x}) = \boldsymbol{U} + \boldsymbol{\Omega} \times \boldsymbol{x} \quad \text{for } \boldsymbol{x} \in \mathcal{S}. \tag{24}$$

$$\boldsymbol{u}(\boldsymbol{x}) = 0 \quad \text{for } |\boldsymbol{x}| \to \infty. \tag{25}$$

Finally, the particle velocities $\boldsymbol{U}$ and $\boldsymbol{\Omega}$ are determined by the conditions of no net force and torque on the particle, accounting for contributions due both to hydrodynamic and electric stresses,

$$\int_{\mathcal{S}} (\boldsymbol{\sigma} + \boldsymbol{\sigma}_e) \cdot \boldsymbol{n} \mathrm{d}\mathcal{S} = 0, \tag{26}$$

$$\int_{\mathcal{S}} \boldsymbol{x} \times (\boldsymbol{\sigma} + \boldsymbol{\sigma}_e) \cdot \boldsymbol{n} \mathrm{d}\mathcal{S} = 0. \tag{27}$$

Here, $\boldsymbol{\sigma} = -p\boldsymbol{\delta} + (\nabla \boldsymbol{u} + \nabla \boldsymbol{u}^T)$ is the hydrodynamic stress tensor and $\boldsymbol{\sigma}_e = \boldsymbol{E}\boldsymbol{E} - \frac{1}{2}E^2\boldsymbol{\delta}$ is the Maxwell stress tensor. This condition follows from the application of the divergence theorem to the Stokes equation (23), noting that $\boldsymbol{f}_e = \nabla \cdot \boldsymbol{\sigma}_e$.

Parameter	Symbol	Value
H_2O_2 concentration[1]	$C^0_{H_2O_2}$	3.03 M
H_2O concentration[1]	$C^0_{H_2O}$	51.6 M
H_2O dissociation constant	K_w	1.82×10^{-16} M
H_2O_2 dissociation constant	K_p	2.40×10^{-12} M
H_2O association rate constant[2]	k_{-w}	1.40×10^{11} M^{-1} s^{-1}
H^+ diffusivity	D_{H^+}	9.31×10^{-9} m^2 s^{-1}
OH^- diffusivity	D_{OH^-}	5.27×10^{-9} m^2 s^{-1}
HO_2^- diffusivity†	$D_{HO_2^-}$	—
H_2O_2 diffusivity[3]	$D_{H_2O_2}$	1.43×10^{-9} m^2 s^{-1}
Surface potential*	Φ_s	-40 mV
**Derived Parameters**		
H_2O_2 association rate constant‡	k_{-p}	1.04×10^{11} M^{-1} s^{-1}
Ionic strength	$C^\infty_\pm$	2.70×10^{-6} M
Characteristic diffusivity	$D_\pm$	5.37×10^{-9} m^2 s^{-1}
Reaction-diffusion length	λ	139 nm
Debye screening length	λ_D	185 nm
Velocity scale	U_0	1.04×10^{-3} m s^{-1}
**Dimensionless Parameters**		
Surface potential	Φ_s	-1.6
Debye screening length	β	1.33
H_2O_2 concentration	γ	1.1×10^6
OH^- concentration	ω	1.3×10^{-3}
H^+ diffusivity	D_{H^+}	1.73
OH^- diffusivity	D_{OH^-}	0.98
H_2O_2, HO_2^- diffusivity	$D_{H_2O_2}, D_{HO_2^-}$	0.27

Supplementary Table 1: Parameter estimates for a 10 wt% H_2O_2 solution in water at 25°C based on data from the CRC Handbook of Physics and Chemistry[4] unless otherwise noted. †This value was unavailable; we therefore set $D_{HO_2^-} = D_{H_2O_2}$. ‡This value was calculated using equation (28) in the main text. *The surface potential is assumed equal to the zeta potential, which was measured previously for Au-Pt nanorods.[5]

Supplementary Note 2: Transport-Limited Regime.

We hypothesize that the reaction parameter μ is sufficiently large that the electrochemical reactions are limited by the transport of HO_2^- to the platinum surface ($\mu \gg 1$). Even in this limit, the reaction does little to reduce the local concentration of peroxide, which is present in great excess ($\gamma \gg 1$). We will therefore assume that the concentration of peroxide is everywhere constant and equal to its bulk value $C_{H_2O_2} = \gamma$. Under these conditions, the model has a well defined solution for the 1D concentration profiles in the vicinity of a planar platinum surface (Fig. 4b). The rate-limiting species HO_2^- is continuously generated in the bulk and diffuses to the surface at a rate

$$\boldsymbol{n} \cdot \boldsymbol{j}_{HO_2^-} = -0.246 \quad \text{(dimensionless)}, \tag{28}$$

$$\boldsymbol{n} \cdot \boldsymbol{j}_{HO_2^-} = -0.246 \frac{D_\pm C_\pm^\infty}{\lambda} = -0.246 D_\pm^{1/2} k_{-p}^{1/2} K_p^{3/4} C_{H_2O_2}^{3/4} \quad \text{(dimensional)}. \tag{29}$$

Concentration variations extend over the reaction-diffusion length λ before approaching the equilibrium bulk values.

This prediction compares favorably with electrochemical measurements of the partial current densities of anodic oxidation and cathodic reduction of hydrogen peroxide on platinum for $C_{H_2O_2}^0 =$ 0.1 M in a 0.5 M K_2SO_4 supporting electrolyte at pH = 5.9.[6] At the mixed potential, the measured currents are ca. 1 A/m^2, which corresponds to a peroxide consumption rate of 1×10^{-5} mol/m^2·s. The above expression predicts a comparable rate of 0.93×10^{-5} mol/m^2·s.

The rate of H_2O_2 decomposition by this electrochemical mechanism represents a small fraction of the overall decomposition rate, which includes both electrochemical and non-electrochemical processes. The overall rate of peroxide decomposition by platinum-polystyrene Janus particles (2 μm diameter) in 10 wt% H_2O_2 (3.0 M) was measured previously to be $(8 \pm 4) \times 10^{10}$ molecules per second per particle (0.02 mol/m^2·s).[7] The model above predicts a rate of 5.1×10^{-5} mol/m^2·s (ca. 400 times smaller).

Using the above results, we can evaluate the approximation that the peroxide concentration is everywhere constant. The overall rate of peroxide decomposition is of order 0.02 mol/m^2·s, which must be balanced by the flux of peroxide to the surface. The diffusive flux of peroxide to a particle of size a is approximately $D_{H_2O_2} \Delta C_{H_2O_2}/a$, where $\Delta C_{H_2O_2}$ is the difference in the peroxide concentration between the surface and the bulk. Equating these two estimates implies a concentration difference of $\Delta C_{H_2O_2} \sim 10$ mM, which is much smaller than the bulk concentration of $C_{H_2O_2} = 3$ M.

Supplementary Note 3: Equilibrium Dissociation of H_2O and H_2O_2.

Far from the particle, the concentrations of H^+, HO_2^-, and OH^- are determined by the equilibrium dissociation of H_2O and H_2O_2. The conditions for dissociation equilibrium are given by

$$K_w = \frac{C_{H^+} C_{OH^-}}{C_{H_2O}} \quad \text{and} \quad K_p = \frac{C_{H^+} C_{HO_2^-}}{C_{H_2O_2}}, \tag{30}$$

where $K_w = 1.82 \times 10^{-16}$ M and $K_p = 2.40 \times 10^{-12}$ M are the dissociation coefficients for water and peroxide, respectively. Additionally, we have the following constraints due to species conservation and electroneutrality,

$$C^0_{H_2O} = C_{H_2O} + C_{OH^-} \tag{31}$$

$$C^0_{H_2O_2} = C_{H_2O_2} + C_{HO_2^-} \tag{32}$$

$$C_{H^+} = C_{OH^-} + C_{HO_2^-}, \tag{33}$$

where $C^0_{H_2O}$ and $C^0_{H_2O_2}$ denote the analytical concentrations of water and peroxide, respectively. For a given peroxide concentration, Supplementary Equations (30)–(33) can be solved to determine the concentrations of the respective dissociation products.

Under the relevant conditions ($C^0_{H_2O_2} \sim 1$ M), the concentrations of water and peroxide are nearly identical to their analytical values—that is, $C_{H_2O} = C^0_{H_2O}$ and $C_{H_2O_2} = C^0_{H_2O_2}$. With this approximation, the ion concentrations can be expressed as

$$C_{H^+} = C^0_{\pm}\sqrt{1+\omega}, \quad C_{HO_2^-} = \frac{C^0_{\pm}}{\sqrt{1+\omega}}, \quad C_{OH^-} = \frac{\omega C^0_{\pm}}{\sqrt{1+\omega}}, \tag{34}$$

where $C^0_{\pm} = (K_p C^0_{H_2O_2})^{1/2}$ is a characteristic concentration scale, and $\omega = K_w C^0_{H_2O} / K_p C^0_{H_2O_2}$ is dimensionless parameter measuring the amount of ions due to water dissociation relative to that of peroxide dissociation. In experiment, this parameter is small $\omega \sim 10^{-3} \ll 1$; below, we assume that $\omega \to 0$.

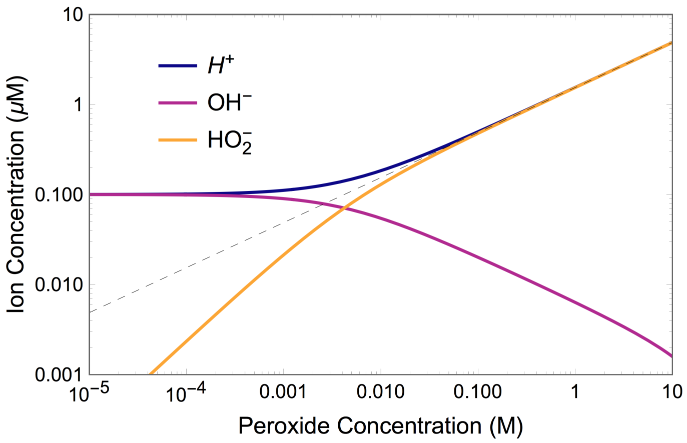

Equilibrium ion concentrations for the self-ionization of hydrogen peroxide in water at 25° C. Here, the peroxide concentration is computed from the peroxide weight fraction using the reported densities of aqueous peroxide solutions.[1]

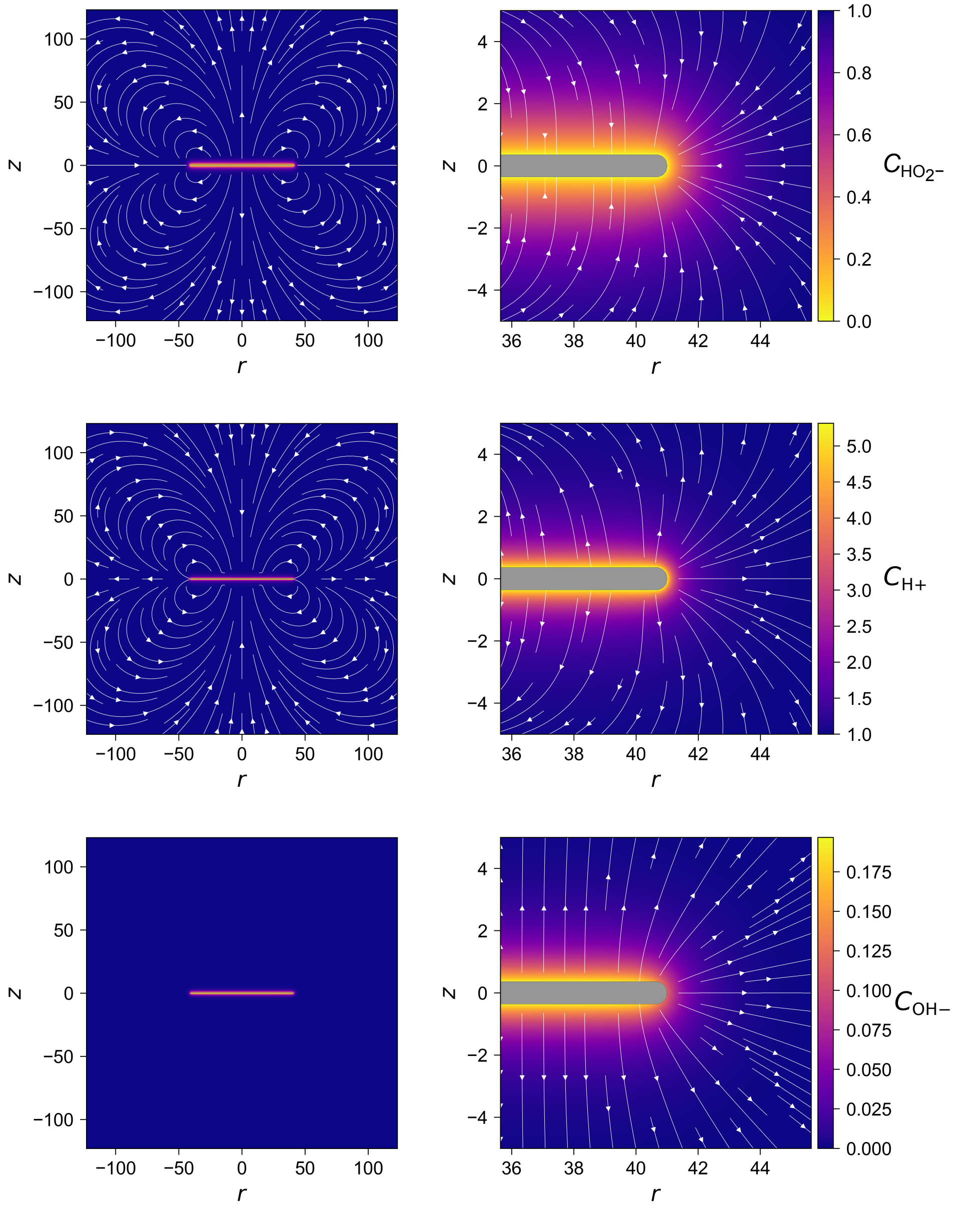

Supplementary Figure 3: Numerical solution for a circular disk. The colormaps show the ion concentrations (scaled by $C_{\pm}^{\infty} = (K_o C^0_{H_2O_2})^{1/2}$) around a platinum disk of radius $a = 41\lambda$ and thickness $d = 0.72\lambda$. Parameter values are listed in Supplementary Table 1. The streamlines denote the respective ion fluxes $\boldsymbol{j}_i$. The ion fluxes are omitted in the far field plot of C_{OH^-} (bottom-left) as the hydroxide concentration and current fall rapidly (exponentially) to zero beyond the reaction-diffusion length λ.

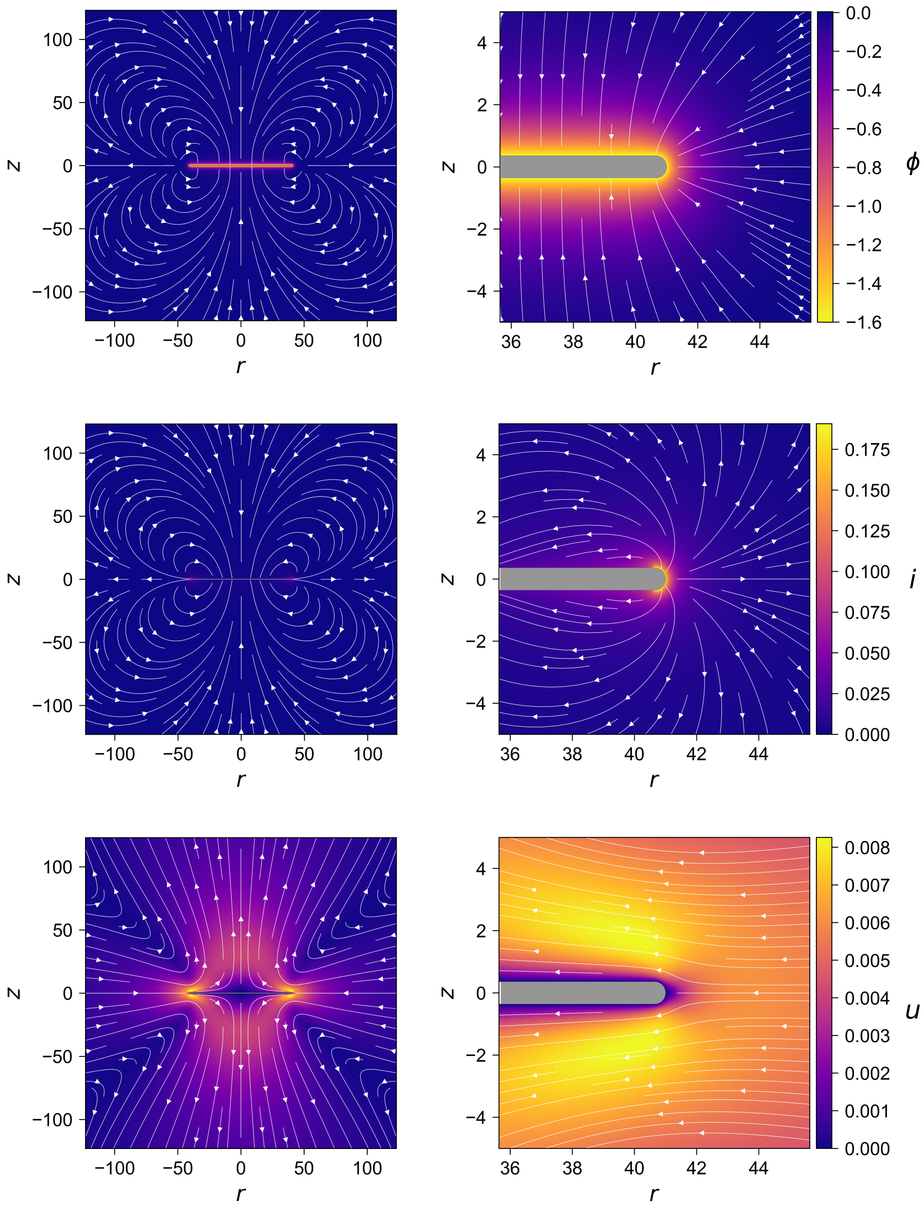

Supplementary Figure 4: Numerical solution for a circular disk. The colormaps show the electric potential (scaled by k_BT/e), the electric current (scaled by $eD_\pm C_\pm^\infty/\lambda$), and the fluid velocity (scaled by $U_0 = C_\pm^\infty k_BT\lambda/\eta$) around a platinum disk of radius $a = 41\lambda$ and thickness $d = 0.72\lambda$. Parameter values are listed in Supplementary Table 1. The streamlines in the top plots denote the electric field $\boldsymbol{E} = -\nabla\phi$.

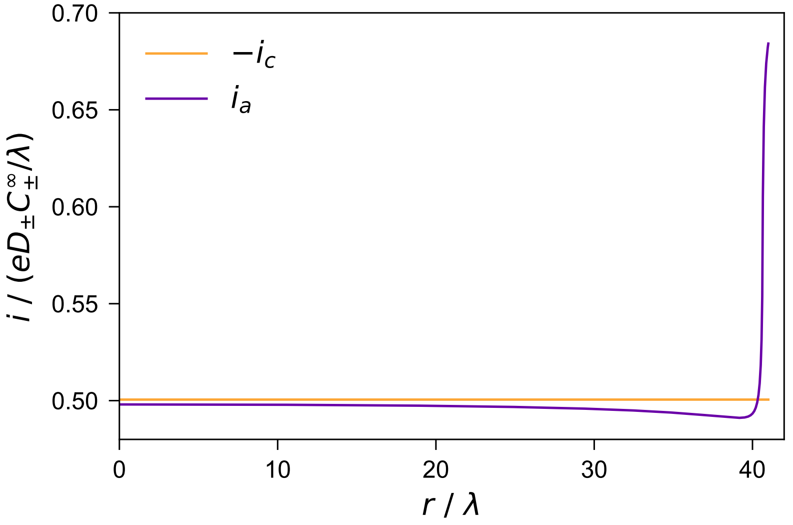

Supplementary Figure 5: Numerical solution for a circular disk. Partial current densities, i_a and i_c, for the anodic and cathodic reactions (scaled by $eD_{\pm}C^{\infty}_{\pm}/\lambda$) as a function of radial position r around a platinum disk of radius $a = 41\lambda$ and thickness $d = 0.72\lambda$. Parameter values are listed in Supplementary Table 1.

Supplementary Note 4: Rotation of Twisted Star Particles.

To estimate the angular velocity $\boldsymbol{\Omega} = \Omega \boldsymbol{e}_z$ of a twisted star particle, we decompose the Stokes flow $\boldsymbol{u}$ around the active particle into two simpler flows denoted $\boldsymbol{u}'$ and $\boldsymbol{u}''$. In the first, the particle rotates with velocity $\boldsymbol{\Omega}' = \Omega \boldsymbol{e}_z$ in the absence of any body force $\boldsymbol{f}' = 0$. In the second, a body force $\boldsymbol{f}'' = \boldsymbol{f}_e$ drives fluid flow around a stationary particle with $\boldsymbol{\Omega}'' = 0$. Owing to the linearity of the Stokes equations, these auxiliary flows can be superimposed to obtain the desired flow, $\boldsymbol{u} = \boldsymbol{u}' + \boldsymbol{u}''$. Each of these flows is associated with a net torque on the particle,

$$\boldsymbol{L} = \int_{\mathcal{S}} \boldsymbol{x} \times (\boldsymbol{\sigma} + \boldsymbol{\sigma}_e) \cdot \boldsymbol{n} \mathrm{d}\mathcal{S}, \tag{35}$$

$$\boldsymbol{L}' = \int_{\mathcal{S}} \boldsymbol{x} \times \boldsymbol{\sigma}' \cdot \boldsymbol{n} \mathrm{d}\mathcal{S}, \tag{36}$$

$$\boldsymbol{L}'' = \int_{\mathcal{S}} \boldsymbol{x} \times (\boldsymbol{\sigma}'' + \boldsymbol{\sigma}_e) \cdot \boldsymbol{n} \mathrm{d}\mathcal{S}, \tag{37}$$

such that the net torque on the active particle can be written as $\boldsymbol{L} = \boldsymbol{L}' + \boldsymbol{L}''$. Owing the symmetry of the particle, all torques are oriented in the z-direction—for example, $\boldsymbol{L} = L\boldsymbol{e}_z$. According to equation (27), the net torque on the active particle is zero, $\boldsymbol{L} = 0$. Moreover, the torque on the rotating particle is linearly related to its velocity as $\boldsymbol{L}' = -R\Omega$, where $R > 0$ is a resistance coefficient. By estimating the torque on the stationary particle L'', we can therefore determine the velocity Ω as

$$\Omega = \frac{L''}{R}. \tag{38}$$

Below we discuss our estimates of the resistance R and the torque $\boldsymbol{L}''$.

Resistance, R**.** In an unbounded fluid, the rotation of a disk-like particle of radius a can be approximated by that of a oblate ellipsoid of equal radius and vanishing thickness, $R = \frac{32}{3}\eta a^3$. In experiment, however, the particles rotate at some height h above a planar substrate. For small separations $h \ll a$, the resistance to rotation is due largely to shear forces acting on the particle's bottom surface $\mathcal{S}_b$

$$R \approx \int_{\mathcal{S}_b} \frac{\eta r}{h} \mathrm{d}\mathcal{S} = \frac{\pi \eta a^4}{2h} \left(1 + 3b^2 + \frac{3}{8} b^4 \right), \tag{39}$$

where b is the dimensionless fin length. For $b \ll 1$, we obtain the approximation used in the main text—namely, $R = \pi \eta a^4 / 2h$.

Torque, L''**.** For thin disk-like particles rotating at a small height h above a planar substrate, the torque (37) can be approximated by neglecting the particle edges and integrating over the particle's top surface $\mathcal{S}_t$

$$L'' = \int_{\mathcal{S}_t} (x\sigma''_{yz} - y\sigma''_{xz}) \mathrm{d}\mathcal{S}. \tag{40}$$

In this approximation, the contribution due to the electric stress is zero for particles with uniform surface potentials, for which $\boldsymbol{n} \cdot \boldsymbol{\sigma}_e = \frac{1}{2} E^2 \boldsymbol{n}$. As noted in the main text, we do not compute the tangent components of the hydrodynamic stress directly. Instead, we compute the radial component of the surface stress σ''_{rz} on a thin circular disk and then map them onto the surface of the twisted star particles. This heuristic approach is discussed in more detail in the following section.

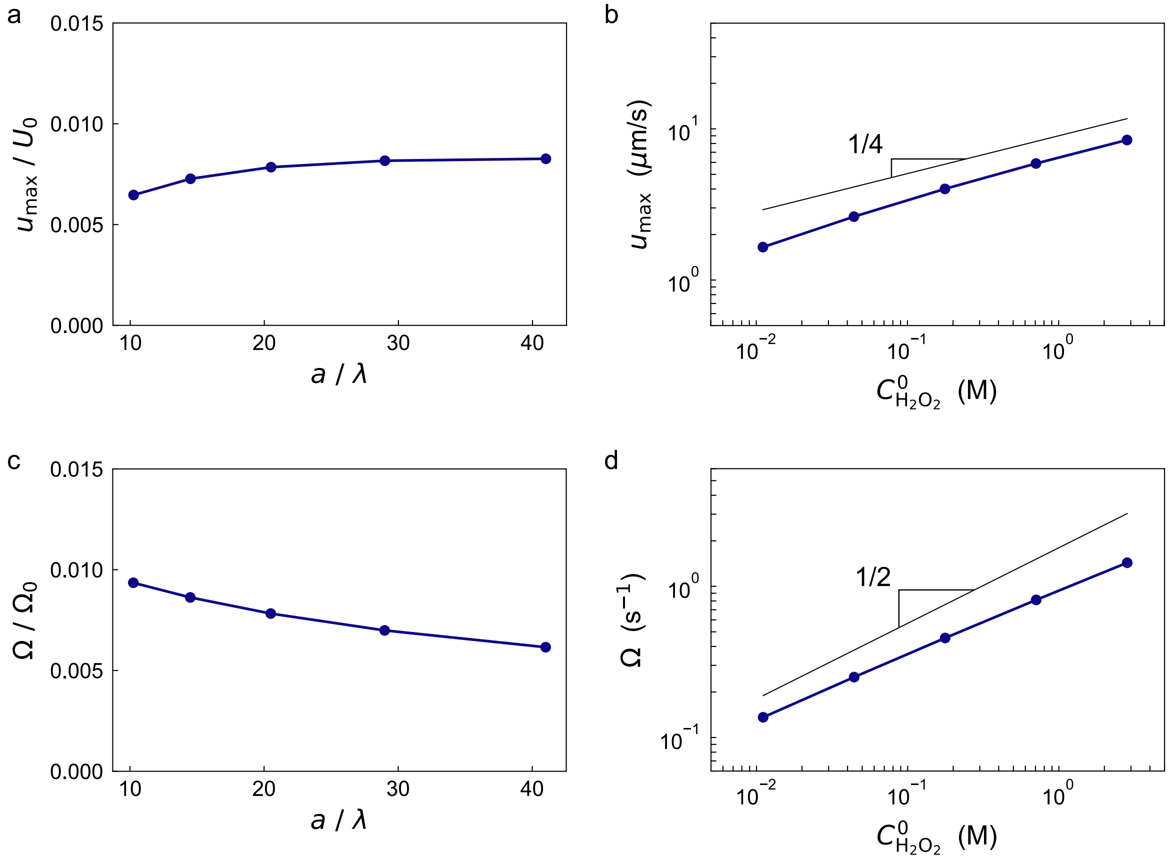

Supplementary Figure 6: Effect of peroxide concentration, $C_{\mathrm{H_2O_2}}$**.** (a) Maximum velocity u_{max} (scaled by $U_0 = C_{\pm}^{\infty} k_B T \lambda / \eta$) around a circular disk as a function of the ratio a/λ. The disk thickness is $\delta = (0.72/41)a$; other parameters are given in Supplementary Table 1. (b) Maximum velocity u_{max} as a function of peroxide concentration as computed from the data in (a) and the parameter estimates in Supplementary Table 1. (c) Rotation velocity Ω (scaled by $\Omega_0 = C_{\pm}^{\infty} k_B T h / \eta a$) for a twisted star particle with $b = 0.3$, $c = 2$, and $n = 4$ computed from the solution in (a). (d) Rotation velocity Ω as a function of peroxide concentration as computed from the data in (c) and the parameter estimates in Supplementary Table 1.

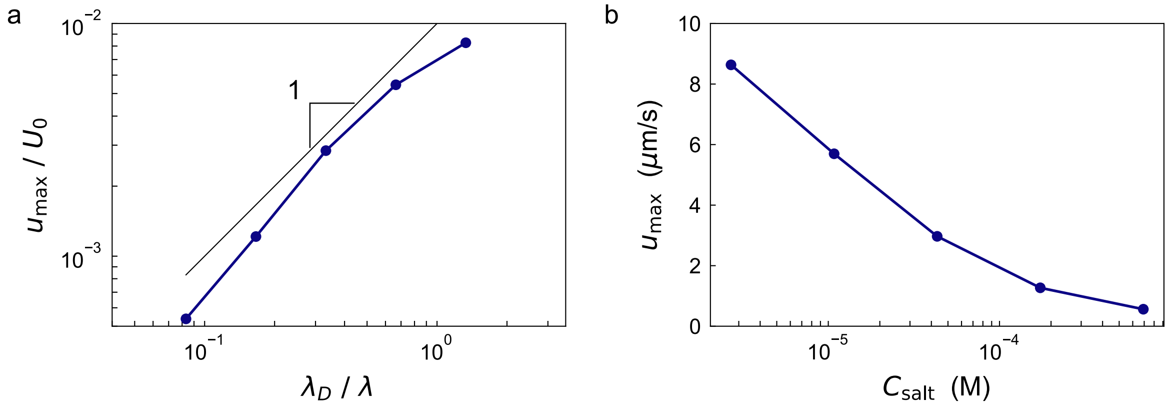

Supplementary Figure 7: Effect of added salt. (a) Maximum velocity $u_{\max}$ (scaled by $U_0 = C_\pm^\infty k_B T\lambda/\eta$) around a circular disk as a function of the parameter $\beta = \lambda_D/\lambda$ appearing in Supplementary Equation (19). The disk has a radius of $a = 41\lambda$ and thickness of $\delta = 0.72\lambda$; other parameters are given in Supplementary Table 1. (b) The data in (a) can be used to approximate the effect of added salt if we allow that $\lambda_D \approx (\varepsilon k_B T/2e^2 C_{\text{salt}})^{1/2}$ where C_{salt} is an effective salt concentration. The maximum flow velocity around a circular disk decreases monotonically with C_{salt} in qualitative agreement with experimental observations. A more careful investigation on the effects of added salt is planned for future work.

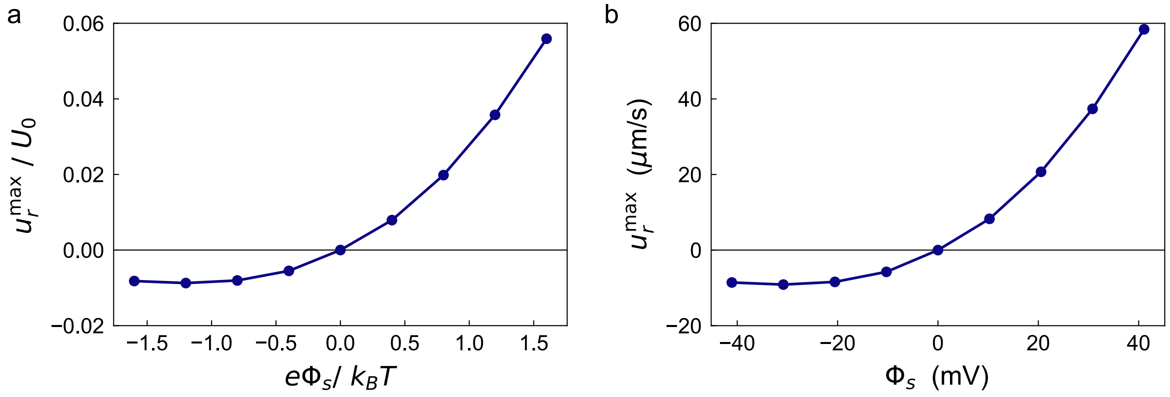

Supplementary Figure 8: Effect of surface potential, Φ_s. (a) Maximum velocity in the radial direction $u_r^{\max}$ (scaled by $U_0 = C_\pm^\infty k_B T\lambda/\eta$) around a circular disk as a function of surface potential Φ_s (scaled by $k_B T/e$). The disk has a radius of $a = 41\lambda$ and thickness of $\delta = 0.72\lambda$; other parameters are given in Supplementary Table 1. (b) Same data as (a) in dimensional units for the parameter values in Supplementary Table 1. The flow velocity is radially inward ($u_r < 0$) for negative surface potentials and radially outward ($u_r > 0$) for positive surface potentials. Note also that positive surface potentials result in larger flows due to the enhanced transport of negatively charge HO_2^- species from solution to the particle surface.

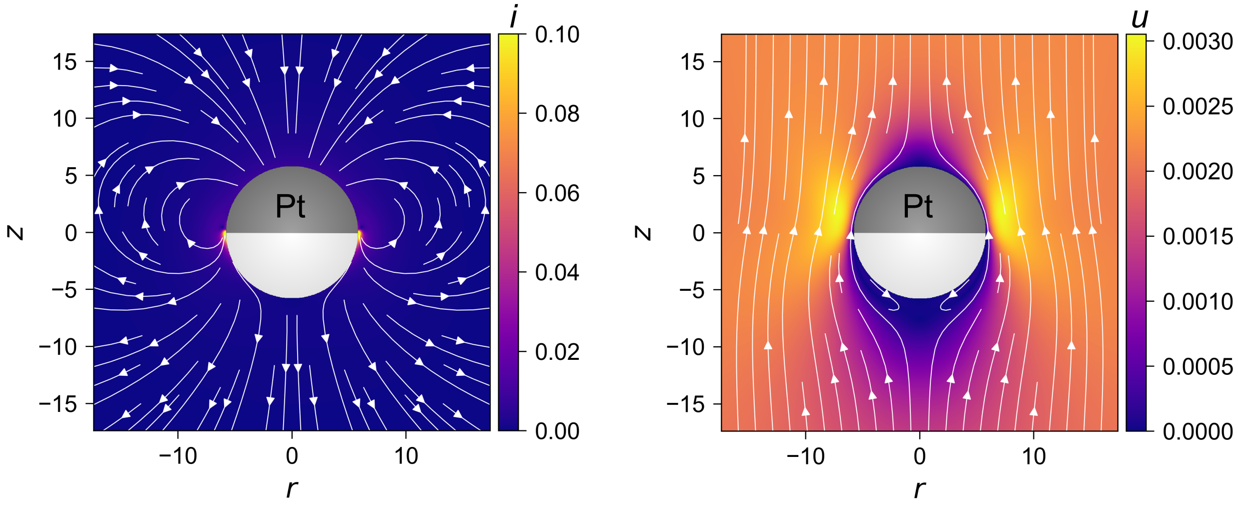

Supplementary Figure 9: Propulsion of a Pt Janus Sphere. Electric current $\boldsymbol{i}$ (left) and fluid velocity $\boldsymbol{u}$ (right) around a Pt Janus sphere computed numerically for (dimensionless) parameters: $a = 5.8$, $\beta = 1.33$, $\Phi_s = -1.6$, $D_{\mathrm{H}} = 1.73$, $D_{\mathrm{OH^-}} = 0.98$, and $D_{\mathrm{HO_2^-}} = 0.27$ as summarized in Supplementary Table 1. Here, lengths are scaled by the reaction diffusion length $\lambda = 140$ nm, current by $eD_{\pm}C_{\pm}^{\infty}/\lambda = 10$ A/m^2, and velocity by $U_0 = C_{\pm}^{\infty}k_BT\lambda/\eta = 1.0$ mm/s. These parameters correspond to the experimental conditions of Howse *et al.*[8] for a polystyrene sphere of radius $a = 0.81$ μm coated on one hemisphere with a 5.5 nm thick layer of platinum. The particle is immersed in an aqueous solution of 10 wt% H_2O_2 ($C_{\mathrm{H_2O_2}} = 3.0$ M or 7.4 vol%). The polystyrene hemisphere is assumed to be chemically inert with a zeta potential equal to that of the platinum hemisphere. The particle moves toward its polystyrene hemisphere with a computed speed of 2.2 μm/s. For comparison, Figure 4a of Howse *et al.* shows an experimental propulsion velocity of ca. 3 μm/s.[8] We emphasize that the model has no free parameters; however, several parameters are uncertain. Notably, an increase in the particle zeta potential could help to reconcile the 40% difference between experiment and theory. Further work is required to investigate the impact of different model parameters on the predicted propulsion velocity.

Supplementary Note 5: Conformal Mapping and the Role of Particle Shape.

Here, we consider how both the particle shape and the stress distribution influence the magnitude and direction of the torque $\boldsymbol{L}''$ that drives particle rotation. For thin particles ($\delta \ll a$), the torque L_z'' is approximated as

$$L_z'' = \int_{\mathcal{S}_t} \boldsymbol{r} \times (\boldsymbol{e}_z \cdot \boldsymbol{\sigma}''(\boldsymbol{r})) \mathrm{d}\mathcal{S}, \tag{41}$$

where $\boldsymbol{r} = x\boldsymbol{e}_x + y\boldsymbol{e}_y$ is the position vector from the center of the particle face, and $\mathcal{S}_t$ is the planar surface on the top side of the particle. To compute the integral, we parameterize the position vector as $\boldsymbol{r}(\rho, s)$ where ρ and s are orthogonal curvilinear coordinates (Supplementary Figure 10). The curve $\rho = 1$ corresponds to the perimeter of the particle, and $\rho = 0$ to the particle origin.

We will assume that the stress depends only on ρ and is oriented along the negative ρ-direction—that is, $\boldsymbol{e}_z \cdot \boldsymbol{\sigma}''(\boldsymbol{r}) = -\sigma''(\rho)\boldsymbol{e}_\rho$ with $\sigma''(\rho) > 0$. This heuristic approximation is exact for the circular disk, for which the coordinates (ρ, s) are identical to the polar coordinates (r, θ). For a weakly twisted star (small b and c), we hypothesize that this approach correctly predicts the direction and the characteristic magnitude of the torque on the particle. We note, however, that the magnitude and/or direction of the force density could vary with position along the perimeter s. Such variations are likely to be small when the reaction-diffusion length λ is much smaller than the radius of curvature of particle perimeter.

As detailed below, we find that the torque may be positive or negative depending on the stress distribution $\sigma''(\rho)$. When the stress is concentrated within a thin region near the edge of the disk, the torque on an R particle is positive, resulting in CCW rotation. When the stress is distributed across a thicker region around the particle parameter, the torque is negative, resulting in CW rotation (as in experiment). These qualitative trends agree with previous studies of similar particles rotating via asymmetric acoustic streaming (see below).[9]

Orthogonal Curvilinear Coordinates (ρ, s)

The orthogonal coordinates $\rho(\boldsymbol{r})$ and $s(\boldsymbol{r})$ satisfy the Cauchy-Riemann equations

$$\frac{\partial \rho}{\partial x} = \frac{\partial s}{\partial y} \quad \text{and} \quad \frac{\partial s}{\partial x} = -\frac{\partial \rho}{\partial y}, \tag{42}$$

which ensures that the unit vectors $\boldsymbol{e}_\rho$ and $\boldsymbol{e}_s$ in the ρ and s directions are everywhere orthogonal. In particular, the unit vectors $\boldsymbol{e}$ and scale factors h are defined in the usual manner as

$$\boldsymbol{a}_s = \frac{\partial \boldsymbol{r}}{\partial s} = h_s \boldsymbol{e}_s, \tag{43}$$

$$\boldsymbol{a}_\rho = \frac{\partial \boldsymbol{r}}{\partial \rho} = h_\rho \boldsymbol{e}_\rho. \tag{44}$$

By construction, the unit vectors satisfy the following conditions to create a right-handed orthogonal coordinate system

$$\boldsymbol{e}_\rho \cdot \boldsymbol{e}_s = 0 \quad \text{and} \quad \boldsymbol{e}_\rho \times \boldsymbol{e}_s = \boldsymbol{e}_z. \tag{45}$$

To construct the coordinate $\rho(\boldsymbol{r})$, we first solve for an auxiliary function $v(\boldsymbol{r})$ that satisfies the Poisson equation on a two-dimensional domain bounded by the particle contour $\mathcal{C}$,

$$\nabla^2 v = 2\pi\delta(\boldsymbol{r}) \quad \text{with} \quad v(\boldsymbol{r}) = 0 \text{ for } \boldsymbol{r} \in \mathcal{C}. \tag{46}$$

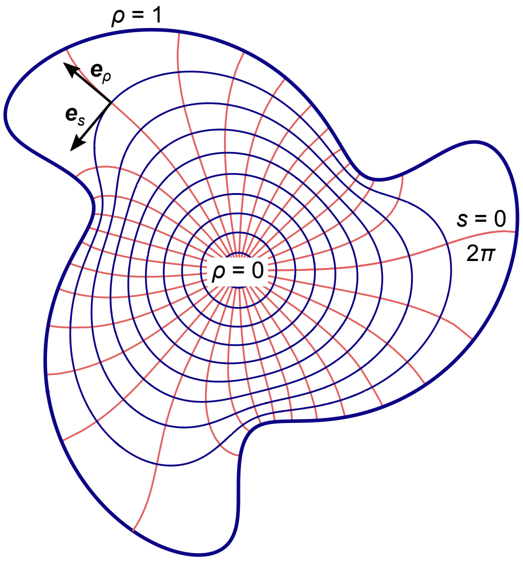

Supplementary Figure 10: Orthogonal curvilinear coordinate system (ρ, s) for a twisted star particle with $c = 1$, $n = 3$, and $b = 0.3$. The particle face corresponds to the region $0 < \rho < 1$ and $0 < s < 2\pi$.

The function $v(\boldsymbol{r})$ is set to zero along the perimeter of the particle, and a point sink is positioned at its origin. For a circular domain of unit radius, this problem can be solved analytically to obtain

$$v(r) = \ln r \quad \text{for } 0 < r \leq 1. \tag{47}$$

Note that the radial coordinate is given by $r = \exp(v)$. For the twisted star domain, the solution $v(\boldsymbol{r})$ can be computed numerically to determine the generalized radial coordinate $\rho(\boldsymbol{r}) = \exp(v(\boldsymbol{r}))$, which is zero at the particle origin and one along its perimeter. Finally, the coordinate $s(\boldsymbol{r})$ can be computed from $\rho(\boldsymbol{r})$ by numerical integration of the Cauchy-Riemann equations (42).

Thick Regions

When the stress is distributed across the whole particle face, the predicted direction of rotation agrees with the experimental observations. In particular we consider the scenario in which the stress is directed radially inward in the negative $\boldsymbol{e}_\rho$ direction with a constant magnitude across the particle face—that is, $\boldsymbol{e}_z \cdot \boldsymbol{\sigma}'' = -\sigma_o \boldsymbol{e}_\rho$ for $0 < \rho < 1$. The coordinate $\rho(\boldsymbol{r})$ is computed numerically on a twisted star domain using Mathematica's finite element solver, and the unit vector $\boldsymbol{e}_\rho$ is computed using Supplementary Equation (44). The torque on the particle is then computed by numerical evaluation of the integral (41) in Mathematica. Supplementary Figure 11 shows the computed torque as a function of the parameters c and n characterizing the shape of the twisted star particles. The qualitative trends are consistent with the experimental observations. The torque (which is proportional to the angular velocity) is negative for positive values of the asymmetry parameter c, increases in magnitude with increasing asymmetry, and is largely insensitive to the number of fins n.

Thin Regions

When the stress is concentrated near the particle perimeter, the direction of rotation reverses. Here, we consider the scenario in which the stress is given by $\boldsymbol{e}_z \cdot \boldsymbol{\sigma}'' = -\sigma_o \boldsymbol{e}_\rho$ for $\rho_o < \rho < 1$, where

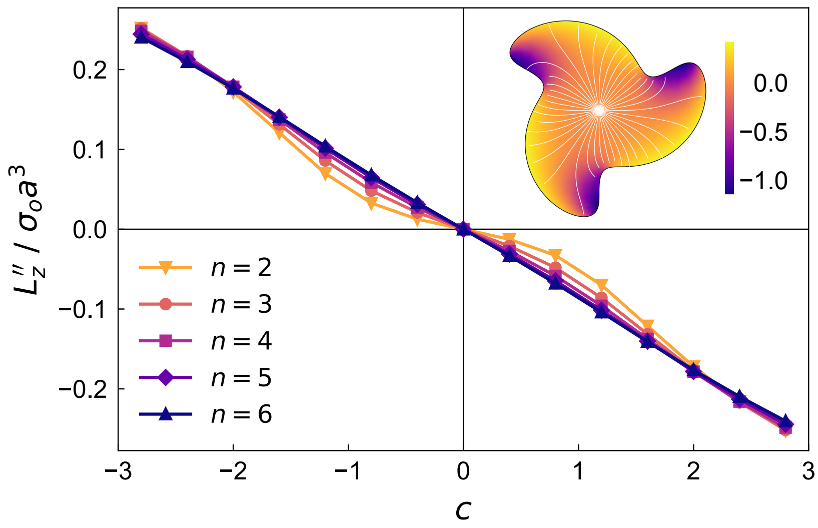

Supplementary Figure 11: Computed torque on a twisted star particle subject to a uniform surface stress in the negative $\boldsymbol{e}_\rho$ direction, $\boldsymbol{e}_z \cdot \boldsymbol{\sigma}'' = -\sigma_o \boldsymbol{e}_\rho$. The torque is plotted as a function of the asymmetry parameter c for different numbers of fins n with $b = 0.3$. The inset shows the torque density (scaled by $\sigma_o a$) on a particle with $c = 1$ and $n = 3$.

the position ρ_o(s) corresponds to a small distance λ from the particle perimeter. In this case, the integral (41) for the torque can be expressed as

$$L_z'' = -\sigma_o \int_0^{2\pi} \int_{\rho_o}^1 (\boldsymbol{r} \times \boldsymbol{e}_\rho) h_\rho h_s \mathrm{d}\rho \mathrm{d}s, \tag{48}$$

where the limit of integration $\rho_o(s)$ is defined implicitly by the condition

$$\lambda = \int_{\rho_o}^1 h_\rho \mathrm{d}\rho. \tag{49}$$

We focus on the limit where the length λ is much smaller than the particle radius a and develop a perturbation theory to compute the torque at successive orders in λ.

Perturbation Theory. The torque is expanded in a Taylor series in λ about $\lambda = 0$

$$L_z'' = \lambda L_z''^{(1)} + \frac{1}{2}\lambda^2 L_z''^{(2)} + \ldots \tag{50}$$

As detailed below, the first order contribution is identically zero; we aim to compute the second order contribution to determine the direction of rotation. To develop this approximation, we will require additional series expansions in the coordinate ρ about $\rho = 1$

$$\boldsymbol{r}(\rho, s) = \boldsymbol{r}^{(0)}(s) + \rho' \boldsymbol{r}^{(1)}(s) + \frac{1}{2}\rho'^2 \boldsymbol{r}^{(2)}(s) + \ldots \tag{51}$$

where $\rho' = \rho - 1$. Here, the zeroth order contribution is simply the particle perimeter introduced in the main text. The first order contribution is the unit vector normal to the particle perimeter. These and other higher order contributions are detailed below. From the expansion (51), we can derive analogous expansions for the coordinate vectors $\boldsymbol{a}$, scale factors h, and unit vectors $\boldsymbol{e}$. Substituting

these expansions into Supplementary Equation (48) for the torque and integrating with respect to ρ, we obtain

$$
\begin{aligned}
L_z'' &= \sigma_o \int_0^{2\pi} \rho_o'(\boldsymbol{r}^{(0)} \times \boldsymbol{a}_\rho^{(0)}) h_s^{(0)} \mathrm{d}s \\
&+ \sigma_o \int_0^{2\pi} \frac{1}{2}\rho_o'^2 \Big((\boldsymbol{r}^{(1)} \times \boldsymbol{a}_\rho^{(0)}) h_s^{(0)} + (\boldsymbol{r}^{(0)} \times \boldsymbol{a}_\rho^{(1)}) h_s^{(0)} + (\boldsymbol{r}^{(0)} \times \boldsymbol{a}_\rho^{(0)}) h_s^{(1)} \mathrm{d}s \Big) + \cdots ,
\end{aligned}
\tag{52}
$$

where $\rho_o' = \rho_o - 1$. To convert this expansion in ρ_o into the desired expansion in λ, we make use of Supplementary Equation (49) to obtain

$$\lambda = -\rho_o' h_\rho^{(0)} - \frac{1}{2}\rho_o'^2 h_\rho^{(1)} + \ldots, \tag{53}$$

This expansion can be inverted to obtain a series expansion for ρ_o' in powers of λ

$$\rho_o' = -\lambda \left(\frac{1}{h_\rho^{(0)}} \right) - \frac{1}{2}\lambda^2 \left(\frac{h_\rho^{(1)}}{h_\rho^{(0)3}} \right) + \ldots \tag{54}$$

Curvilinear Coordinates. We develop a local approximation for the coordinate system (ρ, s) using a series expansion about a point (x_o, y_o) on the particle contour at which $\rho = 1$ and $s = s_o$. We start from the general Taylor expansions

$$\rho(x,y) - 1 = A_{10}x' + A_{01}y' + \frac{1}{2}(A_{20}x'^2 + 2A_{11}x'y' + A_{02}y'^2) + \ldots \tag{55}$$

$$s(x,y) - s_o = B_{10}x' + B_{01}y' + \frac{1}{2}(B_{20}x'^2 + 2B_{11}x'y' + B_{02}y'^2) + \ldots \tag{56}$$

where $x' = x - x_o$ and $y' = y - y_o$. To determine the constants A and B, we require that $s(x, y)$ and $\rho(x, y)$ satisfy the Cauchy-Riemann equations (42). The Cartesian coordinates (x, y) can be expressed in terms of the curvilinear coordinates s and ρ by yet another Taylor expansion

$$x(s,\rho) - x_o = +C_{10}s' + C_{01}\rho' + \frac{1}{2}(C_{20}s'^2 + 2C_{11}s'\rho' + C_{02}\rho'^2) + \ldots \tag{57}$$

$$y(s,\rho) - y_o = D_{10}s' + D_{01}\rho' + \frac{1}{2}(D_{20}s'^2 + 2D_{11}s'\rho' + D_{02}\rho'^2) + \ldots \tag{58}$$

where $s' = s - s_o$. The coefficients C and D can be related to the coefficients A and B by substituting the expansions (57) and (58) into expansions (56) and (55), collecting like powers in s' and ρ', and solving the resulting hierarchy of equations. Notice that the coefficients C_{n0} and D_{n0} can be determined directly by differentiating the parametric equation for the particle perimeter

$$C_{n0} = \left(\frac{\mathrm{d}^n x(s,1)}{\mathrm{d}s^n} \right)_{s=s_o} \quad \text{and} \quad D_{n0} = \left(\frac{\mathrm{d}^n y(s,1)}{\mathrm{d}s^n} \right)_{s=s_o} . \tag{59}$$

The other C and D coefficients are determined implicitly by the Cauchy-Riemann equations (42). The final result of this analysis are the vectors $\boldsymbol{r}^{(n)}(s_o)$ required for Supplementary Equation (51) above

$$\boldsymbol{r}^{(0)} = \begin{bmatrix} x_o \\ y_o \end{bmatrix}, \quad \boldsymbol{r}^{(1)} = \begin{bmatrix} D_{10} \\ -C_{10} \end{bmatrix}, \quad \boldsymbol{r}^{(2)} = \begin{bmatrix} -C_{20} \\ -D_{20} \end{bmatrix} \tag{60}$$

.

Results. For the parametric shapes used here, the first order contribution is identically zero

$$L_z''^{(1)} = -\sigma_o \int_0^{2\pi} (\boldsymbol{r}^{(0)} \times \boldsymbol{a}_\rho^{(0)}) h_s^{(0)} \mathrm{d}s = 0. \tag{61}$$

Physically, this result implies that the application of a uniform force density along the particle perimeter directed normal to the perimeter results in zero net torque on the particle. At second order in λ, the torque has four possible contributions; however, numerical analysis suggests that only one is nonzero

$$L_z''^{(2)} = \sigma_o \int_0^{2\pi} (\boldsymbol{r}^{(0)} \times \boldsymbol{a}_\rho^{(1)}) \frac{h_s^{(0)}}{h_\rho^{(0)2}} \mathrm{d}s \neq 0. \tag{62}$$

Supplementary Figure 12 plots this leading order contribution to the torque as a function of the asymmetry parameter c for different numbers of fins n. Notably, the direction of rotation is opposite of that for the case of thick regions (Supplementary Figure 11) with R particles ($c > 0$) rotating CCW ($\Omega > 0$) and S particles ($c < 0$) rotating CW ($\Omega < 0$).

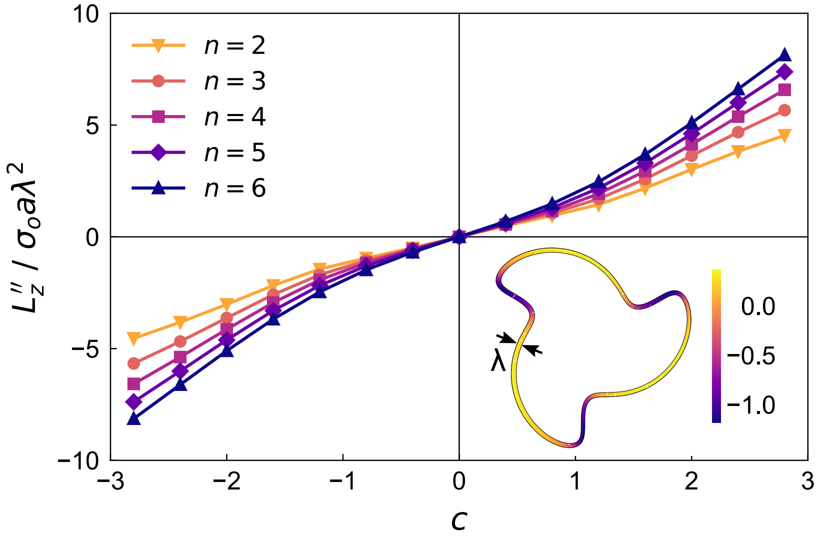

Supplementary Figure 12: Computed torque on a twisted star particle subject to a surface stress in the negative $\boldsymbol{e}_\rho$ direction within a narrow region of thickness λ along the particle perimeter. The torque is plotted as a function of the asymmetry parameter c for different numbers of fins n with $b = 0.3$. The inset shows the torque density (scaled by $\sigma_o a$) on a particle with $c = 1$, $n = 3$, and $\lambda = 0.05a$.

Discussion

To drive particle rotation, a surface force density of the form $\boldsymbol{e}_z \cdot \boldsymbol{\sigma}'' = \sigma''(\rho)\boldsymbol{e}_\rho$ must be distributed over a region of finite thickness. When this region is located along the particle perimeter, the twisted star particles with $c > 0$ are predicted to rotate in the CCW direction (Supplementary Figure 12). This behavior agrees with previous observations of particle rotation driven by acoustic streaming at high frequencies.[9] In that system, the time-averaged force density on the particle was concentrated within a thin region near the particle perimeter and directed inward to the particle center. Interestingly, hydrodynamic simulations at lower frequencies predict that the particle

rotation should reverse direction as the thickness of the viscous boundary layer increases. This prediction is consistent with the above result for thick regions (Supplementary Figure 11).

For the Pt-spinners, the stress distribution lies somewhere between these limiting scenarios. Supplementary Figure 13 shows the stress computed numerically for the circular disk. Although the stress is largest near the perimeter of the disk, there are significant contributions across the entire face. Consequently, when the torque is computed using this distribution (as in Figure 4 of the main text), the direction of rotation agrees with that of the uniform stress.

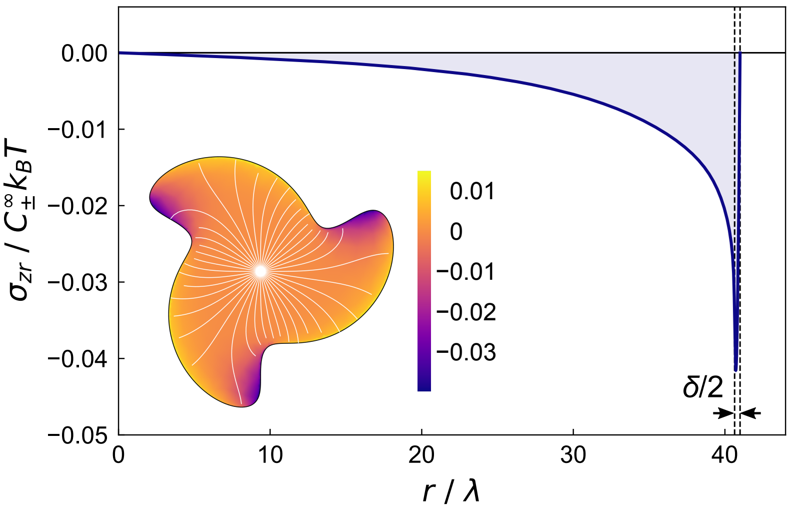

Supplementary Figure 13: Computed stress $\boldsymbol{e}_z \cdot \boldsymbol{\sigma} = \sigma_{zr}(r)\boldsymbol{e}_r$ on a circular disk of radius $a = 41\lambda$ and thickness $\delta = 0.72\lambda$ as a function of the radial position r (scaled by the reaction diffusion length λ). The inset shows the torque density (scaled by $C_{\pm}^{\infty} k_B T a$) on a twisted star with $b = 0.3$, $c = 1$, and $n = 3$ obtained by mapping the stress from the circular disk as $\sigma_{z\rho}(\rho)\boldsymbol{e}_\rho = \sigma_{zr}(r)\boldsymbol{e}_r$. The rounded edge of the disk of width $\delta/2$ is omitted in the mapping.

Supplementary References

1. Easton, M., A.G.Mitchell & Wynn-Jones, W. F. K. The behaviour of mixtures of hydrogen peroxide and water Part 1: Determination of the densities of mixtures of hydrogen peroxide and water. *Trans. Faraday Soc.* **48,** 796–801 (1952).
2. Stillinger, F. H. Proton Transfer Reactions and Kinetics in Water. *Theor. Chem. Adv.* **3,** 177–234 (1978).
3. Van Stroe-Biezen, S. A. M., Everaerts, F. M., Janssen, L. J. J. & Tacken, R. A. Diffusion coefficients of oxygen, hydrogen peroxide and glucose in a hydrogel. *Anal. Chim. Acta* **273,** 553–560 (1993).
4. Haynes, W. M. *CRC handbook of chemistry and physics* (CRC press, 2014).
5. Paxton, W. F., Baker, P. T., Kline, T. R., Wang, Y., Mallouk, T. E. & Sen, A. Catalytically induced electrokinetics for motors and micropumps. *J. Am. Chem. Soc.* **128,** 14881–14888 (2006).
6. Vetter, K. J. *Electrochemical Kinetics* pp. 635–642 (Academic Press, New York, 1967).
7. Brown, A. & Poon, W. Ionic effects in self-propelled Pt-coated Janus swimmers. *Soft Matter,* 4016–4027 (2014).
8. Howse, J. R., Jones, R. A. L., Ryan, A. J., Gough, T., Vafabakhsh, R. & Golestanian, R. Self-Motile Colloidal Particles: From Directed Propulsion to Random Walk. *Phys. Rev. Lett.* **99,** 048102 (2007).
9. Sabrina, S., Tasinkevych, M., Ahmed, S., Brooks, A. M., Olvera de la Cruz, M., Mallouk, T. E. & Bishop, K. J. M. Shape-Directed Microspinners Powered by Ultrasound. *ACS Nano* **12,** 2939–2947 (2018).
