# Supplementary material for: Shape-directed rotation of homogeneous micromotors via catalytic self-electrophoresis
Source: Nat Commun. 2019 Jan 30;10:495. doi: 10.1038/s41467-019-08423-7 (PMC6353883; doi:10.1038/s41467-019-08423-7)
Supplement: Supplementary file 3 — Description of Additional Supplementary Files [file 41467_2019_8423_MOESM3_ESM.pdf]

Description of Additional Supplementary Files

Supplementary Movie 1: Chemically-Powered Shape-Directed Rotation
This movie shows the steady rotation of platinum disks with twisted star shapes in 10 wt% hydrogen peroxide (Figure 1c). The shape of particles is specified by the following parameters: average radius *a* = 5.77 μm, thickness δ = 100 nm, number of fins *n* = 3, asymmetry parameter *c* = 2, and dimensionless fin length *b* = 0.3. Playback is in real time; particles rotate clockwise with a mean speed of $\Omega = 1.5 \pm 0.2$ rad s^{-1}. Scale bar is 20 μm.

Supplementary Movie 2: Shape-Directed Rotation in Particle Mixtures
This movie the steady rotation of platinum disks of different shapes in 10 wt% hydrogen peroxide (Figure 1d). Particles of different shapes rotate in different directions and at different rates in a common environment. Particles differ in the number of fins (n = 2, 3, 4) and the chiral asymmetry (*c* = –2, 0.5, –1, respectively); the average radius *a* = 5.77 μm, thickness δ = 100 nm, and dimensionless fin length *b* = 0.3 are the same for all particles. Playback is in real time. Scale bar is 20 μm.

Supplementary Movie 3: Rotation Reversal in CTAB Solutions
This movie the steady rotation of platinum disks with twisted star shapes in a solution containing 10 wt % hydrogen peroxide and 0.33 mM of the cationic surfactant CTAB (Figure 3b). The shape of particles is specified by the following parameters: average radius *a* = 5.77 μm, thickness δ = 100 nm, number of fins *n* = 3, asymmetry parameter *c* = –2, and dimensionless fin length *b* = 0.3. Particles rotate clockwise. Playback is sped up four times. Scale bar is 20 μm.
